# Emerging trends of therapy related myeloid neoplasms following modern cancer therapeutics in the United States

**DOI:** 10.1038/s41598-021-02497-4

**Published:** 2021-12-02

**Authors:** Abhay Singh, Megan M. Herr, Elizabeth A. Griffiths, Amanda Przespolewski, Mark G. Faber, Chebli Mrad, Eunice S. Wang, Theresa Hahn, Swapna Thota

**Affiliations:** 1 Department of Medicine, Roswell Park Comprehensive Cancer Center, Buffalo, NY 14263 USA; 2 Present Address: Department of Hematology and Medical Oncology, Cleveland Clinic, Taussig Cancer Institute, 10201 Carnegie Avenue, Cleveland, OH 44106 USA; 3 Department of Medicine-Hematology, University of Tennessee Health Science Center, Memphis, TN USA; 4 Department of Hematology and Medical Oncology, Cleveland Clinic Florida, Weston, FL USA; 5 Roswell Park Comprehensive Cancer Center, Elm and Carlton Street, Buffalo, NY 14203 USA

**Keywords:** Cancer, Cancer epidemiology, Haematological cancer

## Abstract

Clonal hematopoiesis (CH) is a risk factor for the development of therapy-related myelodysplastic syndromes (tMDS) and acute myeloid leukemia (tAML). Adoption of targeted-immunotherapeutics since 2011, may alter the risk of CH progression to tMDS/AML. To study this, we evaluated risk of tMDS and tAML in 667 588 ≥ 1-year survivors of non-small cell lung cancer (NSCLC), renal cell carcinoma (RCC), melanoma and multiple-myeloma (MM) diagnosed during: 2000–2005, 2006–2010 and 2011–2016. The risk of tMDS increased significantly after NSCLC across all time periods (P_trend_ = 0.002) while tAML risk decreased from 2006–2010 to 2011–2016, coinciding with increasing use of non-chemotherapeutic agents. tAML risk after RCC decreased (P_trend_ = 0.007) whereas tMDS risk did not significantly change over time. After melanoma, tMDS and tAML risks were similar to the general population. tMDS and tAML risk after MM increased from the first to second time-period, however, only risk of tMDS decreased during last period. We report diverging trends in the risk of tAML and tMDS after adoption of modern cancer therapies for specific cancers. It is imperative to further explore impact of contemporary treatment strategies on clonal evolution. Modern treatments via their discrete mechanism of actions on pre-existing CH may alter the risk of subsequent tMDS and tAML**.**

## Introduction

Therapy-related myelodysplastic syndromes (tMDS) and acute myeloid leukemia (tAML) arise from exposure to cytotoxic chemotherapy and/or radiotherapy^1,2^. The 10-year cumulative incidence of these conditions ranges from 1–10% for different cancers and different chemo-radiotherapy regimens^3,4^. A large population-based registry study reported the risk for development of tMDS/AML to be almost fivefold higher in cancer survivors than the general population^5^. The highest risks may be related to specific cytotoxic agents used within certain regimens, e.g. exposure to alkylating agents for Hodgkin’s lymphoma confers prolonged and six-fold higher risk than the general population^5^. Previous studies have focused on quantifying the impact of known leukemogenic agents such as alkylating agents, topoisomerase II inhibitors or platinum compounds^5–7^. Trends for development of tMDS/AML with modern therapies as well as trends in chemotherapy resistant tumors such as renal cell carcinoma (RCC) and melanoma remain unexplored.

Modern targeted and immune therapeutics for cancer have revolutionized the field of oncology. Specifically, treatments for non-small cell lung cancer [NSCLC; small molecule pathway inhibitors^8,9^ and immune checkpoint inhibitors]^10^, RCC [small molecule and immune checkpoint inhibitors]^11^, melanoma [immunotherapy with cytokines, checkpoint inhibitors and small molecule inhibitors]^12^ and multiple myeloma [MM; immunomodulatory drugs (IMiDs) such as lenalidomide^13^, proteasome inhibitors and monoclonal antibodies]^14^ have evolved considerably (Supplemental Table and sFig. 1). These modern therapies have well described immunomodulatory properties^15^.

Drugs that improve immune surveillance [immune checkpoint inhibitors (ICIs), IMiDs and tyrosine kinase inhibitors (TKIs)] and agents that target certain myeloid disease-associated molecular aberrations, have the potential to halt clonal progression and therefore, decrease tMDS/AML risk after first primary malignancy. Using a large, nationally representative dataset, we aimed to study the risk of developing tMDS and tAML following diagnoses of NSCLC, cutaneous melanoma, RCC, and MM. These cancers were selected since recent and substantial changes in standard treatment have resulted in shifts from the upfront use of cytotoxic regimens to the widespread adoption of targeted and immunomodulatory agents.

## Methods

Eligible subjects included adults (age 20–84 years) diagnosed with first primary NSCLC, RCC, cutaneous melanoma and MM from 2000–2016 from 17 Surveillance, Epidemiology, and End Results Program (SEER) registries. SEER covers approximately 34% of the United States population and captures data on demographics, cancer sequence (e.g. first primary cancer, second primary cancer), and initial course of treatment. The primary cancers were identified using International Classification of Disease for Oncology, 3rd edition (ICD-O-3) morphology and topography codes (Supplemental Table 2). Subjects who survived ≥ 1 year were included and followed for 5 years or through 2017, whichever occurred first. tMDS and tAML were similarly defined by ICD-O-3 morphology and topography codes (Supplemental Table 2), typically defined by </≥ 20% blasts in the bone marrow^16^. We analyzed rates of tMDS/tAML as they are classified together per WHO. Noticing, a diverging trends among these entities we investigated separately due to conceivable heterogeneity in their clonal architecture^17,18^. Data on tMDS progression to tAML are not available in the SEER database.

### Statistical analyses of tMDS and tAML

tMDS and tAML risks were calculated using standardized incidence ratios (SIRs) and 95% confidence intervals (CIs) in SEER*Stat (version 8.3.6). Exact Poisson methods were used to calculate P-values for trends and heterogeneity. SIRs were calculated by dividing the observed number of MDS and AML cases by the number of expected MDS and AML cases in the general population. The expected number of MDS, AML cases were calculated by multiplying the stratified general population incidence rates [by 5-year age groups, race, sex, and calendar year of diagnosis (2000–2005, 2006–2010, 2011–2017)] by the person-time at risk of the cohort^19^. Subjects were followed to earliest of: tMDS or tAML diagnosis, last follow-up, age 85 years, death, 5 years, or end of study (December 31, 2017). In order to reduce surveillance bias, we excluded tMDS and tAML cases that occurred in the first year of follow-up due to increased surveillance and those patients over the age of 85 years due to decreased surveillance. Additionally, we conducted a sensitivity analysis truncating follow-up on December 31, 2012 allowing for 5 years of follow-up to reduce survival bias in the most recent cohort.

### Multivariable Poisson models by first primary cancer

**Figure 1:**
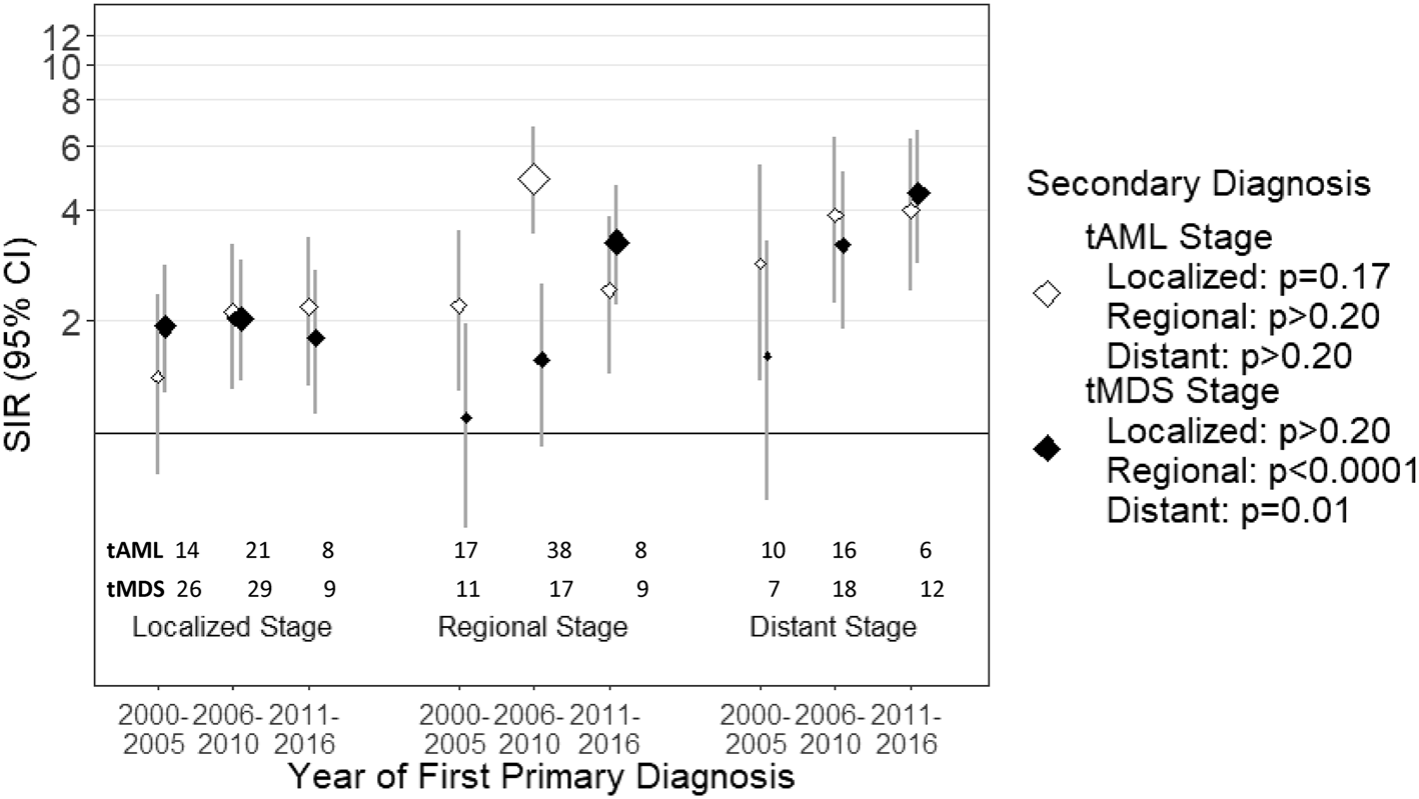
Risk of tMDS and tAML after NSCLC by stage and time period of NSCLC diagnosis. *CI* confidence interval, *NSCLC* non-small cell lung carcinoma, *SIR* standardized incidence ratio, *tMDS* treatment-related myelodysplastic syndrome, *tAML* treatment-related acute myeloid leukemia. ‘tAML’ and ‘tMDS’ inside the figure represents number of patients diagnosed with t-AML or tMDS, in the respective periods.

**Figure 2:**
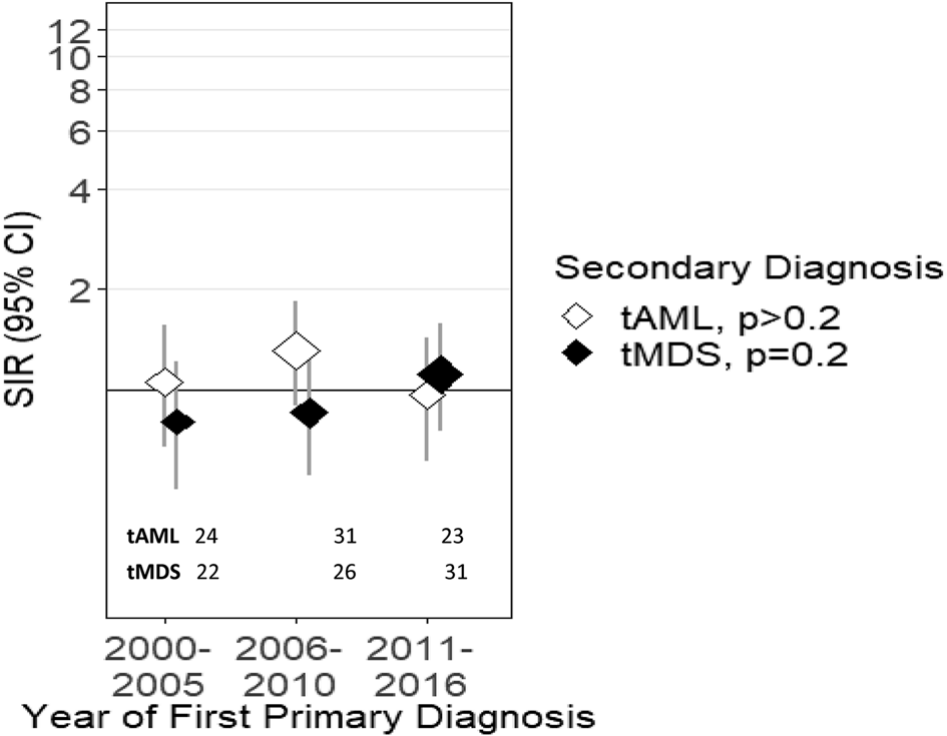
Risk of tMDS and tAML after cutaneous melanoma by time period of cutaneous melanoma diagnosis. *CI* confidence interval, *SIR* standardized incidence ratio, *tMDS* treatment-related myelodysplastic syndrome, *tAML* treatment-related acute myeloid leukemia. ‘tAML’ and ‘tMDS’ inside the figure represents number of patients diagnosed with t-AML or tMDS, in the respective periods.

**Figure 3:**
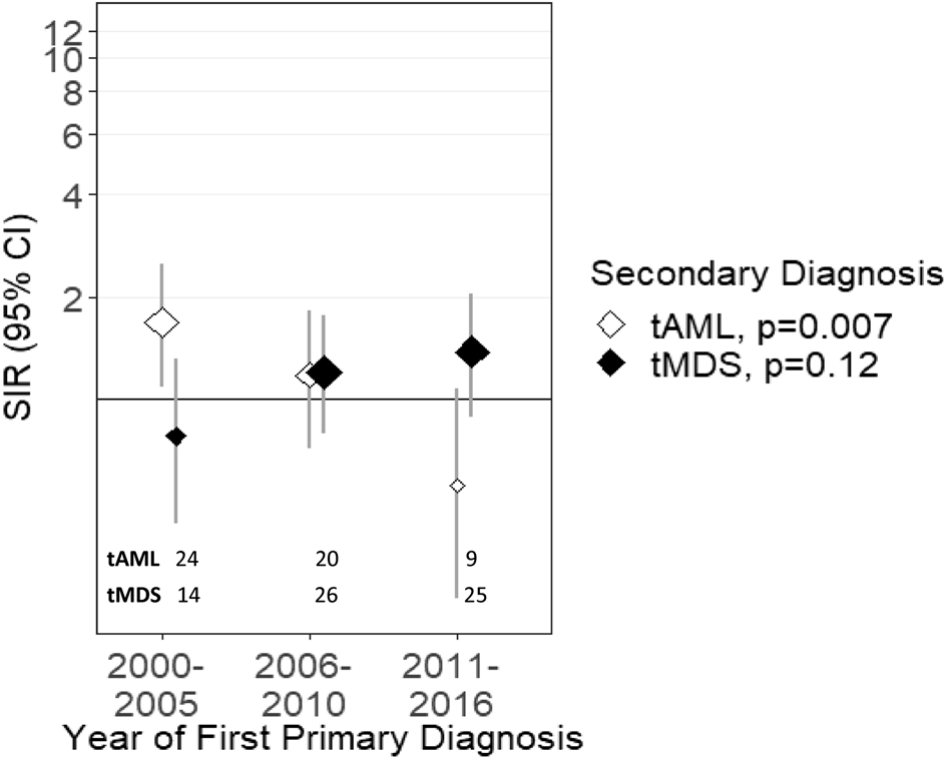
Risk of tMDS and tAML after RCC by time period of RCC diagnosis. *CI* confidence interval, *RCC* renal cell carcinoma, *SIR* standardized incidence ratio, *tMDS* treatment-related myelodysplastic syndrome, *tAML* treatment-related acute myeloid leukemia. ‘tAML’ and ‘tMDS’ inside the figure represents number of patients diagnosed with t-AML or tMDS, in the respective periods.

**Figure 4:**
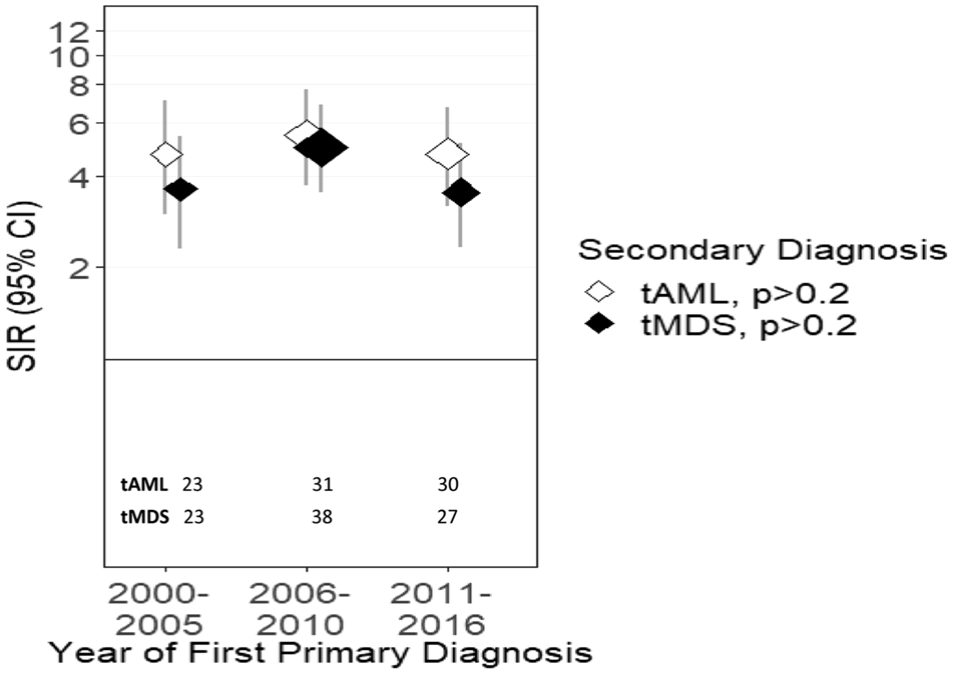
Risk of tMDS and tAML after MM by time period of MM diagnosis. *CI* confidence interval, *MM* multiple myeloma, *SIR* standardized incidence ratio, *tMDS* treatment-related myelodysplastic syndrome, *tAML* treatment-related acute myeloid leukemia. ‘tAML’ and ‘tMDS’ inside the figure represents number of patients diagnosed with t-AML or tMDS, in the respective periods.

To assess for SIR trends and heterogeneity by age, sex, race, chemotherapy, radiation, and calendar time period, we conducted multivariable Poisson models by first primary cancer (Epicure version 2.00.02, Risk Sciences International^20^. Specifically, P_trends_ and P_heterogeneity_ values for Tables 2, 3, Supplemental Tables 3, 4 and P_trends_ in Figs. 1, 2, 3 and 4 were computed using these methods. Models were adjusted for age, sex, and race. The log of the expected number of cases was included as an offset to further indirectly adjust for attained age and calendar year^21^. Two-sided P values to test for heterogeneity and trend were computed using a likelihood ratio test derived from Poisson regression models comparing models with and without the factor of interest. Additional therapy after first-line is not captured by SEER. For all statistical analyses, P < 0.05 was considered statistically significant using two-sided statistical tests.

### Relative survival

Five-year relative survival was calculated to assess if differences in survival across time periods influenced tMDS and tAML development. Relative survival compares all-cause overall survival to a “similar” theoretical cancer-free group^22^. Further details regarding relative survival methodology can be found in the supplementary materials.

## Results

Patient characteristics of 667,588 first primary NSCLC (N = 222,246), cutaneous melanoma (N = 208,154), RCC (N = 132,199) and MM (N = 49,513), initially diagnosed during 2000–2016 and followed through 2017 are shown in Table 1. Patients with first primary cutaneous melanoma tended to be younger (mean age 57 years) and white (98.7%), whereas 21.1% of MM patients were black and 6.5% other races. In analysis of latency (Table 1), median time to develop tMDS after NSCLC, melanoma, RCC and MM were 3.8, 5.0, 5.4 and 3.8 years respectively; similar to median time to develop tAML i.e., 3.6, 5.6, 5.3 and 4.9 respectively. Results of tMDS and tAML risks individually for each primary cancer and across time periods (2000–2005, 2006–2010, 2011–2016) are discussed below.

**Table 1:** Patient characteristics, by initial neoplasm, of ≥ 1-year adult (≥ 20 years old) survivors in 17 SEER registries, 2000–2017.

Characteristic	NSCLC	Cutaneous Melanoma	RCC	Multiple Myeloma
	n = 222 246	n = 208 154	n = 132 199	n = 49 513
	%	%	%	%
**Age at initial diagnosis, years**				
20–39	1.2	14.3	5.4	1.8
40–49	5.7	17.2	14.0	8.7
50–59	19.7	23.7	26.5	22.8
60–69	34.3	23.2	29.6	31.8
70–79	31.9	16.9	20.1	27.5
80–83	7.3	4.8	4.4	7.5
Mean age at initial diagnosis, years	66.1	57.0	60.8	64.6
Median latency for MDS/AML, years	3.8	5.4	5.3	4.3
Median latency for MDS, years	3.8	5.0	5.4	3.8
Median latency for AML, years	3.6	5.6	5.3	4.9
**Sex**				
Male	49.8	55.7	53.7	54.7
Female	50.2	44.3	46.3	45.3
Race				
White/unknown	81.0	98.7	82.7	72.5
Black	11.4	0.4	11.3	21.1
Other	7.6	0.8	6.0	6.5
**Year of first primary malignancy diagnosis**				
2000–2005	32.4	31.4	27.2	28.4
2006–2010	29.8	29.5	30.5	28.6
2011–2016	37.8	39.1	42.2	43.0

17 SEER registries (Atlanta, Georgia; Connecticut; Detroit, Michigan; Hawaii; Iowa; New Mexico; San Francisco-Oakland, Los Angeles, and San Jose-Monterey, California; Seattle-Puget Sound, Washington; Utah; Kentucky; Louisiana; New Jersey, and areas of Rural Georgia, Greater Georgia, and Greater California). Demographics refer to patients with ≤ 5 year latency, except for median latency.

*NSCLC* non-small cell carcinoma, *RCC* renal cell carcinoma, *SEER* Surveillance, Epidemiology and End Results Program.

### Non-small cell lung cancer

Elevated tAML risk was observed after a diagnosis of NSCLC (SIR = 2.68, 95% CI = 2.30–3.11; Table 2). This elevated risk differed by stage of disease (localized: SIR = 1.79, 95% CI = 1.29–2.41; regional: SIR = 3.34, 95% CI = 2.57–4.27; stage IV or distant: SIR = 3.36, 95% CI = 2.30–4.75; Fig. 1). Evaluating the risk across each time period, the risk for tAML after a diagnosis of regional NSCLC increased from 2000–2005 (SIR = 2.21) to 2006–2010 (regional: SIR = 4.89) and then declined in the most recent time period (2011–2016; SIR = 2.42; P_trend_ > 0.20; Fig. 1). Risk for tAML after a diagnosis of localized and distant NSCLC increased across the first two time periods and then remained the same (localized: P_trend_ = 0.17; distant: P_trend_ > 0.2). After conducting sensitivity analyses limiting patients’ diagnoses to on or before December 31, 2012 to limit bias in the most recent cohort, risk of tAML after distant NSCLC decreased in the most recent cohort (SIR = 3.02, 95% CI = 1.11–6.58), similar to tAML risk after regional stage NSCLC patients (Supplemental Table 3).

**Table 2:** Standardized incidence ratios for tAML by age, sex, race, and initial diagnosis year among ≥ 1-year adult first primary NSCLC, melanoma, RCC and MM survivors in 17 SEER registries, 2000–2017.

Characteristic	NSCLC			Cutaneous melanoma			RCC			MM		
	n = 222,246			n = 208,154			n = 132,199			n = 49 513		
	O	SIR	95% CI	O	SIR	95% CI	O	SIR	95% CI	O	SIR	95% CI
Overall	176	2.68*	(2.30, 3.11)	78	1.10	(0.87, 1.37)	53	1.13	(0.84, 1.47)	84	4.99*	(3.98, 6.18)
**Age at first primary neoplasm, years**												
< 50	12	12.59*	(6.50, 21.99)	6	1.11	(0.41, 2.42)	[10]	1.14	(0.54, 2.09)	5	11.59*	(3.76, 27.05)
50–59	31	5.77*	(3.92, 8.20)	17	1.73*	(1.01, 2.78)				21	11.30*	(6.99, 17.27)
60–69	49	2.35*	(1.74, 3.11)	26	1.20	(0.79, 1.76)	14	0.87	(0.48, 1.46)	29	5.35*	(3.58, 7.68)
70–79	77	2.35*	(1.85, 2.93)	23	0.81	(0.51, 1.24)	22	1.14	(0.72, 1.73)	[29]	3.26*	(2.13, 4.56)
≥ 80	7	1.24	(0.50, 2.55)	6	1.09	(0.40, 2.37)	7	2.40	(0.96, 4.94)			
P_trend_	< 0.001			0.10			0.16			< 0.001		
**Sex**												
Male	99	2.57*	(2.09, 3.13)	56	1.11	(0.84, 1.45)	33	0.98	(0.68, 1.38)	52	4.76*	(3.55, 6.24)
Female	77	2.84*	(2.24, 3.55)	22	1.07	(0.67, 1.62)	20	1.49	(0.91, 2.30)	32	5.42*	(3.71, 7.65)
P_heterogeneity_	> 0.20			> 0.20			> 0.20			> 0.20		
**Race**												
White/unknown	140	2.45*	(2.06, 2.89)	77	1.10	(0.86, 1.37)	47	1.14	(0.84, 1.52)	69	5.12*	(3.99, 6.48)
Black	25	5.14*	(3.33, 7.59)	[< 5]	1.80	(0.05, 10.00)	[6]	1.03	(0.38, 2.23)	10	3.87*	(1.86, 7.12)
Other	11	3.03*	(1.51, 5.42)							5	6.32*	(2.05, 14.76)
P_heterogeneity_	0.02			na			> 0.20			> 0.20		
**Initial diagnosis year**												
2000–2005	42	1.93*	(1.39, 2.60)	24	1.06	(0.68, 1.57)	24	1.69*	(1.08, 2.51)	23	4.74*	(3.01, 7.12)
2006–2010	75	3.38*	(2.65, 4.23)	31	1.27	(0.87, 1.81)	20	1.18	(0.72, 1.83)	31	5.46*	(3.71, 7.75)
2011–2016	59	2.73*	(2.08, 3.53)	23	0.96	(0.61, 1.45)	9	0.57	(0.26, 1.07)	30	4.75*	(3.21, 6.78)
P_trend_	0.10			> 0.20			0.007			> 0.20		
**Chemotherapy**												
Any chemotherapy	78	1.82*	(1.44, 2.27)	75	1.07	(0.84, 1.34)	51	1.12	(0.83, 1.47)	17	2.56*	(1.49, 4.10)
No/unknown chemotherapy	98	4.30*	(3.49, 5.24)	< 5	5.33*	(1.10, 15.57)	< 5	1.30	(0.16, 4.69)	67	6.56*	(5.09, 8.33)
P_heterogeneity_	< 0.001			na			na			< 0.001		
**Radiation**												
Any radiation	87	1.86*	(1.49, 2.29)	75	1.07	(0.84, 1.34)	51	1.10	(0.82, 1.45)	72	5.08*	(3.97, 6.40)
No/unknown radiation	89	4.72*	(3.79, 5.81)	< 5	3.86	(0.80, 11.27)	< 5	3.01	(0.36, 10.88)	12	4.50*	(2.33, 7.87)
P_heterogeneity_	< 0.001			na			na			> 0.20		

*MM* multiple myeloma, *NSCLC* non-small cell lung carcinoma, *O* observed, *RCC* renal cell carcinoma, *SEER* Surveillance, Epidemiology and End Results Program, *SIR* standardized incidence ratio, *tAML* treatment-related acute myeloid leukemia, *95% CI* 95% confidence interval.

*P < 0.05.

P values to test differences in the SIRs were computed using a likelihood ratio test derived from Poisson regression models stratified by age at first primary neoplasm, sex, race, initial diagnosis year, and stage of NSCLC. Categories with < 5 observations were not specified to maintain patient confidentiality. P-values do not include categories with < 5.

The risk for development of tMDS was elevated after a diagnosis of NSCLC (SIR = 2.16, 95% CI = 1.86–2.49; Table 3). This risk remained elevated after all stages of NSCLC (localized: SIR = 1.95, regional: SIR = 1.48, distant: SIR = 3.01; Fig. 1) and significantly increased across all the time periods tested (2000–2005: SIR = 1.64, 2006–2010: SIR = 2.05, 2011–2016: SIR = 2.84; P_trend_ = 0.002; Table 3).

**Table 3:** Standardized incidence ratios for tMDS by age, sex, race, and initial diagnosis year among ≥ 1-year adult first primary NSCLC, melanoma, RCC and MM survivors in 17 SEER registries, 2000–2017.

Characteristic	NSCLC			Cutaneous Melanoma			RCC			MM		
	n = 222,246			n = 208,154			n = 132,199			n = 49,513		
	O	SIR	95% CI	O	SIR	95% CI	O	SIR	95% CI	O	SIR	95% CI
Overall	187	2.16*	(1.86, 2.49)	79	0.92	(0.73, 1.14)	65	1.13	(0.87, 1.43)	88	4.06*	(3.26, 5.01)
**Age at first primary neoplasm, years**												
< 60	13	3.24*	(1.73, 5.54)	[29]	0.94	(0.63, 1.35)	8	1.52	(0.66, 3.00)	16	10.99*	(6.28, 17.85)
60–69	59	2.66*	(2.03, 3.43)				24	1.41	(0.90, 2.09)	40	6.91*	(4.93, 9.40)
70–79	98	1.95*	(1.58, 2.37)	45	1.00	(0.73, 1.33)	27	0.90	(0.59, 1.30)	26	2.20*	(1.44, 3.23)
≥ 80	17	1.66	(0.97, 2.66)	5	0.49	(0.16, 1.15)	6	1.12	(0.41, 2.44)	6	2.30	(0.84, 5.00)
P_trend_	0.01			> 0.20			> 0.20			< 0.001		
**Sex**												
Male	111	2.11*	(1.74, 2.54)	52	0.81	(0.60, 1.06)	47	1.12	(0.82, 1.49)	60	4.17*	(3.18, 5.37)
Female	76	2.23*	(1.76, 2.79)	27	1.24	(0.82, 1.81)	18	1.14	(0.68, 1.80)	28	3.86*	(2.56, 5.57)
P_heterogeneity_	> 0.20			0.10			> 0.20			> 0.20		
**Race**												
White/unknown	158	2.08*	(1.76, 2.43)	78	0.91	(0.72, 1.14)	48	0.94	(0.69, 1.25)	72	4.10*	(3.21, 5.17)
Black	14	2.32*	(1.27, 3.89)	[< 5]	1.58	(0.04, 8.82)	12	2.73*	(1.41, 4.76)	[16]	3.90*	(2.23, 6.33)
Other	15	3.29*	(1.84, 5.42)				5	2.12	(0.69, 4.94)			
P_heterogeneity_	> 0.20			na			0.01			> 0.20		
**Initial diagnosis year**												
2000–2005	47	1.64*	(1.20, 2.18)	22	0.80	(0.50, 1.21)	14	0.78	(0.43, 1.31)	23	3.61*	(2.29, 5.42)
2006–2010	64	2.05*	(1.58, 2.62)	26	0.85	(0.55, 1.24)	26	1.20	(0.79, 1.76)	38	4.98*	(3.52, 6.83)
2011–2016	76	2.84*	(2.23, 3.55)	31	1.11	(0.75, 1.57)	25	1.38	(0.89, 2.03)	27	3.53*	(2.32, 5.13)
P_trend_	0.002			0.20			0.12			> 0.20		
**Chemotherapy**												
Any chemotherapy	93	1.60^#^	(1.29, 1.96)	77	0.90	(0.71, 1.12)	64	1.14	(0.88, 1.46)	27	2.98^#^	(1.96, 4.34)
No/unknown chemotherapy	94	3.30*	(2.67, 4.04)	< 5	3.33	(0.40, 12.03)	< 5	0.55	(0.01, 3.06)	61	4.84*	(3.70, 6.22)
P_heterogeneity_	< 0.001			na			na			> 0.20		
**Radiation**												
Any radiation	101	1.62*	(1.32, 1.97)	78	0.92	(0.72, 1.14)	64	1.12	(0.87, 1.43)	73	3.96*	(3.10, 4.98)
No/unknown radiation	86	3.53*	(2.83, 4.36)	< 5	1.02	(0.03, 5.69)	< 5	1.27	(0.03, 7.05)	15	4.66*	(2.61, 7.69)
P_heterogeneity_	< 0.001			na			na			> 0.20		

*MM* multiple myeloma, *NSCLC* non-small cell lung carcinoma, *O* observed, *RCC* renal cell carcinoma, *SEER* Surveillance, Epidemiology and End Results Program, *SIR* standardized incidence ratio, *tMDS* treatment-related myelodysplastic syndrome, *95% CI* 95% confidence interval.

*P < 0.05.

P values to test differences in the SIRs were computed using a likelihood ratio test derived from Poisson regression models stratified by age at first primary neoplasm, sex, race, initial diagnosis year, and stage of NSCLC. Categories with < 5 observations were not specified to maintain patient confidentiality. P-values do not include categories with < 5.

### Melanoma

After a diagnosis of first primary cutaneous melanoma, there was no increased risk for development of tAML in the time period between 2000–2016 (SIR = 1.10, 95% CI = 0.87–1.37, Table 2). Risk for the development of tAML across each of the time periods tested did not demonstrate any specific trend (2000–2005: SIR = 1.06, 2006–2010: SIR = 1.27, 2011–2016: SIR = 0.96; P_trend_ > 0.2; Table 2, Fig. 2). Similarly, there were no trends in the risk for tMDS development after a melanoma diagnosis across and within these time periods (SIRs = 0.80, 0.85, 1.11; Table 3, Fig. 2; P_trend_ = 0.2).

### Renal cell carcinoma

The risk for development of tAML after a first primary RCC was elevated in the first time period (2000–2005: SIR = 1.69) and subsequently declined across each time periods investigated thereafter (P_trend_ = 0.007). Most recently, tAML risk after RCC was lower than general population suggesting a non-significant protective effect (2011–2016: SIR = 0.57, Fig. 3, Table 2). In contrast, tMDS risk after RCC demonstrated a non-significant increase over time (SIRs = 0.78, 1.20, 1.38, P = 0.12, Table 3, Fig. 3).

### Multiple myeloma

tAML risk after first primary MM did not significantly change across time periods (P_trend_ > 0.2; Table 2, Fig. 4), despite the change from chemo-based regimens to immunomodulatory regimens. When the initial MM diagnosis year was limited to 2012 to allow for a full 5 years of follow-up, tAML risk in the most recent time period increased considerably (SIR = 7.26, 95% CI = 4.49–11.09) although the trend remained non-significant (P_trend_ = 0.18; sTable 3).

tMDS risk also did not significantly change over time (SIRs = 3.61, 4.98, 3.53; P_trend_ > 0.2; Table 3, Fig. 4), despite the change from chemo-based regimens to immunomodulatory regimens. When the initial MM diagnosis year was limited to 2012 to allow for a full 5 years of follow-up, tMDS risk was lower in the most recent time period than previous years (SIR = 2.27, 95% CI = 0.98–4.47; sTable 4), in contrast to tAML risk after MM.

### Combined tMDS/tAML risk

We investigated the combined risk for tMDS/AML among 565,149 adults initially diagnosed with NSCLC, melanoma, RCC and MM during 2000–2016 (followed up through 2017), sTable 5.

Based on a total of 810 cases, tMDS/tAML occurred more often than expected in NSCLC (SIR 2.38; 2.14–2.63) and MM (SIR 4.46; 3.81, 5.17). The combined risk increased significantly after NSCLC across the three time periods (P_trend_ < 0.001) but not after MM (> 0.20). After initial chemotherapy for NSCLC and MM, combined tMDS/AML risk was elevated (< 0.001). For both combined melanoma and RCC, despite adequate number of observed tMDS/tAML cases (157 and 118 respectively), no particular trends were noted (SIRs similar to general population), speaking to the non-conventional cancer therapy approaches used in the treatment of these cancers.

### Multivariate Poisson regression models to evaluate observed differences in SIRs

In analyses by age, SIRs for tMDS and tAML were highest among patients diagnosed with first primary NSCLC and MM at younger ages (P_trend_ ≤ 0.01 for all; Tables 2 and 3). In analyses by race, risk for tAML after NSCLC and tMDS after RCC was highest among those of black race compared to white/unknown and other races (tAML after NSCLC: P_heterogeneity_ = 0.02;Table 2, tMDS after RCC: P_heterogeneity_ = 0.01; Table 3). No differences in risk existed for tAML or tMDS by sex. Chemotherapy and/or radiotherapy exposure significantly heightened risks for tMDS after NSCLC as well as tAML after NSCLC compared with no therapy. The risk for tAML after MM in patients initially treated with chemotherapy was significantly elevated (P_heterogeneity_ < 0.001); however, risk for tMDS did not differ by initial chemotherapy treatment (P_heterogeneity_ > 0.2). There were too few patients who received chemotherapy or radiation as a part of their initial therapy to evaluate risk of tAML or tMDS after RCC or melanoma.

### Survival

**Figure 5:**
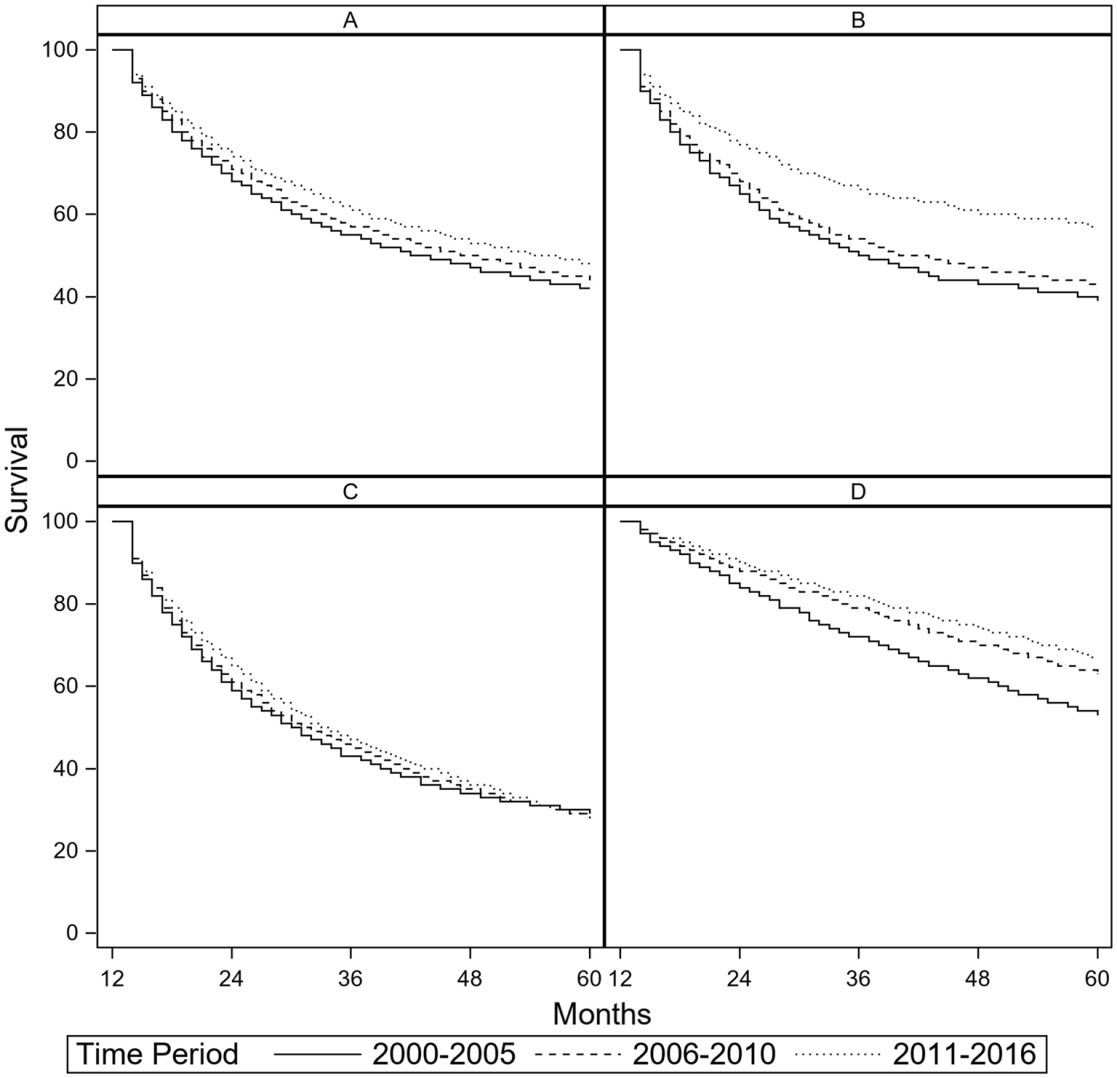
Relative survival (RS) by cancer type and by time period (**A**) RS after non-small cell lung carcinoma by time period, (**B**) RS after advanced melanoma by time period, (**C**) RS after advanced renal cell carcinoma by time period, and (**D**) RS after multiple myeloma by time period.

5-year relative survival of patients who survived their first year differed by cancer type: advanced RCC had the lowest 5-year relative survival (29%), followed by NSCLC (45%), advanced melanoma (48%) and MM (61%). Five-year survival increased across all three time periods for NSCLC, advanced melanoma and MM (NSCLC: 42%, 44%, and 48%, Fig. 5A; advanced melanoma: 39%, 43%, and 57%, Fig. 5B; MM: 53%, 63%, and 67%, Fig. 5D). Five-year survival after advanced RCC has remained stable across all three figure periods (29%, 28% and 29%, Fig. 5C).

## Discussion

With substantial shifts in treatments over the last two decades, we have demonstrated for the first time changes in tMDS and tAML risk over time that highlights distinctions between tMDS and tAML trends. Specifically, tAML risk decreased after NSCLC, and RCC in the most recent time period and increased after myeloma. Additionally, an increase in tMDS risk after NSCLC, as well as RCC and decrease after MM over time was noted. The following factors could contribute to these trends: (i) Decreasing utilization of leukemogenic agents that are specifically associated with tAML (ii) underlying molecular uniqueness of tMDS and tAML which may be differentially sensitive to various targeted and immune agents (iii) host specific factors such as germline predisposition, smoking etc., unaccounted in this study but randomly controlled by comparison to general population (iv) relative survival changes over time. Additionally, improved case ascertainment of MDS diagnosis in SEER registry over time would not explain these trends, as it would lead to consistent increase in tMDS after all tumor types over progressive time periods. Similar to prior studies^5,6^, we noted that combined tMDS/tAML risk continues to be high after specific cancers, NSCLC and MM. This effect is likely driven by increased tMDS risk after NSCLC and tAML risk after MM, as discussed below.

The concordance of the timeline of novel therapy adoption (sTable 1) and changing trends of tMDS and tAML is intriguing and perhaps reflects impact of these agents on myeloid clones that preexist before disease presentation. Mutational profile of tAML has overlap with AML arising from antecedent myeloid neoplasia^23^. While there is a certain overlap of molecular make up between these two entities, key distinctions between tAML and tMDS remain to be explored; an active area for continued research. Mutations of RAS pathway genes (*PTPN11, NRAS, KRAS, FLT3*) are predominant in tAML in comparison to tMDS wherein mutations such as *TET2, ASXL1, SRSF2* and *SF3B1* are common^17,18^. Mutations in these genes (pre-tAML clones) have been reported to predate overt myeloid neoplasms with increasing variant allele frequencies (VAFs) over time, from baseline to diagnosis^4,24,25^. Several widely applied targeted agents (sunitinib, sorafenib, everolimus, vemurafenib, erlotinib etc.) act via RAS pathways^26^ and could potentially influence clonal propagation of these pre-malignant tAML clones. Since, RAS pathway mutations are observed primarily in tAML, it is plausible that the mutational variability between tMDS and tAML might have partly contributed to the opposing tAML and tMDS risk trends. In myeloma cohort the risk trends differed, with higher tAML risks over time and lower tMDS risk. Treatment with IMID has shown impact on CH genes (*TET2, ASXL1, SRSF2 and SF3B1*)^27^, and potentiation of *TP53* clone, leading to increased tAML risk^28^.

Risk of both tMDS and tAML after early stage NSCLC (localized and regional) showed a steep increase in the second period (2006–2010). A rapid adaptation of adjuvant chemotherapy with platinum agents in the treatment of early stage lung cancers during the first period (2000–2005) may directly explain this trend^29–32^. In the most recent period (2011–2016), we noted a decline in tAML risk after early stage (regional but not localized) NSCLC. Targeted therapies (*EGFR/ALK*-TKIs) and ICIs have revolutionized treatment of advanced NSCLC and about 25% of NSCLC patients can harbor EGFR and ALK mutations^9^, but these were not approved for early stage NSCLC during periods of our study. Therefore, decrease in tAML risk after regional stage NSCLC in the most recent period is rather surprising. It is expected that a high proportion of these early stage NSCLCs (especially, regional) have distant recurrences over time^33^ and go on to receive either TKIs and ICIs that increase tumor surveillance. Localized NSCLCs have lower likelihood of distant recurrence^33^ when compared to regional NSCLCs and hence, are less likely to be recipients of newer therapies. This would explain lack of downtrend in tAML risk after localized stages (Fig. 1).

Studies conducted thus far have been limited by the smaller number of observed tMDS/AML cases after melanoma and RCC or due to lack of receipt of chemotherapy^5–7^. For RCC, chemotherapy use was common until the approval of VEGF TKIs in 2005. With decreasing chemotherapy use and introduction of targeted therapies and ICIs, a potential downtrend in tAML risk is evident in 2011–2016 after RCC and melanoma; however, an uptrend is emerging for tMDS risk. Unique disease biology of tAML; for instance, *FLT3* [an important tAML^18^ and pre-tAML^4^ clone] is a known target for sorafenib and sunitinib^34^ and used extensively for RCC treatment^11^.

Historically, MM patients had the highest risk for tAML^5^. With decreasing use of alkylators, this risk was declining by the end of last century; however, more recently, the risk increased^5,35^, after the introduction of novel agents (e.g. IMiDs and proteasome inhibitors). Similar to previous SEER studies, we observed continued increase in tAML risk as stem cell transplantation and lenalidomide continue to remain a cornerstone of myeloma therapy^5,36^. A selective advantage of the *RAS* pathway gene mutations as well as *TP53* clones^28,37,38^ may have contributed to an increased risk for tAML after MM. It was interesting to note declining tMDS risk after MM. Recent epidemiological studies have noted that overall MDS incidence has been declining since 2011^39^, which may in part explain the trend noted. Alternatively, this observation may align with report on impact of IMiDs on CH^27^. Both these findings were consistent with our multivariate Poisson regression modeling which reaffirmed that risk for tAML after MM in patients initially treated with chemotherapy was significantly elevated (sTable 5); whereas, risk for tMDS did not differ by initial chemotherapy treatment.

We report new emerging trends of myeloid neoplasms since the advent of targeted and immunotherapies. In addition to above, it is key to consider possibility of surveillance bias in survivors of first primary cancers due to increased surveillance and monitoring of peripheral blood count. It is debatable if this would lead to distinctions observed in the type of secondary myeloid cancers. tMDS and tAML are often classified together^16^; in our report we highlight their unique population trends. Limitations include the lack of data on specific chemotherapy, immunotherapy or targeted drugs and treatment patterns of recurrent/progressive disease. It is important to acknowledge that not all newly-diagnosed AML/MDS patients with prior solid malignancy including those with chemo-and radiotherapy exposure will have therapy-“related” disease. There likely are other factors at play such as germline predisposition, smoking etc., which are unaccounted in this study. Additionally, to make the time periods comparable, we truncated follow-up in the first two time periods to 5 years. The most recent time period had shorter follow-up, which could bias our results towards the null. However, if a bias existed, we would anticipate a similar trend for both tMDS and tAML.

In conclusion, we present unique and diverging trends of tMDS and tAML after a first primary cancer diagnosis in the era of modern cancer therapeutics. The underlying reasons are multifactorial and concordance of these trends with modern therapeutics is intriguing. With an increased understanding of the characteristics of CH and its evolution to tMDS/AML when exposed to external stressors, our future research focus remains on delineating the interaction of cancer therapies and pre-leukemic clones.

## Supplementary Information

Supplementary Information.

## Data Availability

All data are publicly available using SEER*Stat. Presented in part in the poster discussion session at the 2020 ASCO Virtual Scientific Program.
