# Supplementary material for: Emerging trends of therapy related myeloid neoplasms following modern cancer therapeutics in the United States
Source: Sci Rep. 2021 Dec 2;11:23284. doi: 10.1038/s41598-021-02497-4 (PMC8639740; doi:10.1038/s41598-021-02497-4)
Supplement: Supplementary file 1 — Supplementary Information. [file 41598_2021_2497_MOESM1_ESM.docx]

**Supplementary Materials**

Emerging trends of therapy related myeloid neoplasms following modern cancer therapeutics in the United States

| Supplemental Table 1.  Population-based patterns of decreasing chemotherapy use and adoption of modern therapies for non-small cell lung cancer, cutaneous melanoma, renal cell carcinoma and multiple myeloma before and after year 2000 | | | | |
| --- | --- | --- | --- | --- |
| **First primary malignancy** | **Before 2000** | **2000-2005** | **2006-2010** | **2011-2016** |
| *Non-small cell lung cancer* | 1990s- Platinum backbone chemotherapy, gemcitabine, vinorelbine, taxanes^4,5,6^ | Early 2000s- Adjuvant chemotherapy adoption for most early stage NSCLCs^7,8,9^  2003/04 – Gefitinib^10^ and erlotinib^11^ FDA approval | 2010- Crizotinib effective in ALK+ NSCLC^12^ | 2012 – Immunotherapy in clinical trials^13^  2014- Crizotinib effective in ROS+ NSCLC^13^  2015- Immunotherapy superior to docetaxel in second line^13^ |
| *Cutaneous melanoma* | 1970-90s- Dacarbazine^16^  1996- Interferon α-2b^15^  1998 – High dose interleukin-2^15^ |  | Pegylated interferon α-2b^15^ | Dacarbazine use stopped with approvals of Ipilimumab and Vemurafenib in 2011^17,18^  2013- Dabrafenib and trametinib^15, 16^  2014- Pembrolizumab^15, 16^  2015- Nivolumab plus ipilimumab^15,16^ |
| *Renal Cell Carcinoma* | 1990-1998 -Numerous chemotherapeutic agents in trials and practice^19^  1992- High dose interleukin-2^19^ | December 2005- Sorafenib^20,21^ | January 2006 – Sunitinib^20,21^  2007 – Temsirolimus^20,21^  2009 – Everolimus^21^  2009 – Pazopanib^21^ | 2012- Axitinib^20,21^  2015 – Nivolumab^20,21^ |
| *Multiple Myeloma* | 1970s- Melphalan plus prednisone^1^  1984 – Vincristine, Adriyamycin, Dexamethasone (VAD)^2^  1999 – Thalidomide^3^ (FDA approval 2006) | 2003 – Bortezomib^14^ | 2006- Thalidomide^14^  2006- Lenalidomide^14^ | 2012- Carfilzomib^14^  2013 – Pomalidomide^14^  2014 –Post-transplant maintenance lenalidomide^14^  2015 – Panobinostat^14^  2015 – Ixazomib^14^  2015 – Daratumumab^14^  2015 – Elotozumab^14^ |

**References for Supplemental Table 1.**

1. Alexanian R, Haut A, Khan U, et al. Treatment for multiple myeloma, combination chemotherapy with different melphalan dose regimens. JAMA. 1969;208:1680-1685.2
2. Alexanian R, Barlogie B, Tucher S. VAD-based regimens as primary treatment for multiple myeloma. Am J Hematol. 1990;33:86-89
3. Singhal S, Mehta J, Desikan R, Ayers D, Roberson P,Eddlemon P, Munshi N, Anaissie E, Wilson C, Dhodapkar M, Zeldis J, Barlogie B, Siegel D, Crowley J. Antitumor activity of thalidomide in refractory multiple myeloma . N Engl J Med. 1999;341:1565-1571.
4. Lilenbaum RC, Herndon JE, List MA, et al: Single-agent versus combination chemotherapy in advanced non-small-cell lung cancer: The Cancer and Leukemia Group B (study 9730). J Clin Oncol 23:190-196, 2005.
5. Wozniak AJ, Crowley JJ, Balcerzak SP, et al: Randomized trial comparing cisplatin with cisplatin plus vinorelbine in the treatment of advanced non-small cell lung cancer: A Southwest Oncology Group study. J Clin Oncol 16:2459-2465, 1998.
6. Sandler AB, Nemunaitis J, Denham C, et al: Phase III trial of gemcitabine plus cisplatin versus cisplatin alone in patients with locally advanced or metastatic non-small-cell lung cancer. J Clin Oncol 18:122-130, 2000.
7. Scagliotti GV, Fossati R, Torri V, et al: Randomized study of adjuvant chemotherapy for completely resected stage I, II, or IIIA non-small cell lung cancer. J Natl Cancer Inst 95:1453-1461, 2003.
8. International Adjuvant Lung Cancer Trial Collaborative Group: Cisplatin-based adjuvant chemotherapy in patients with completely resected non-small cell lung cancer. N Engl J Med 350:351-360, 2004.
9. Winton T, Livingston R, Johnson D, et al: Vinorelbine plus cisplatin vs observation in resected non-small-cell lung cancer. N Engl J Med 352:2589-2597, 2005.
10. Cohen MH, Williams GA, Sridhara R, Chen G, Pazdur R. FDA drug approval summary: gefitinib (ZD1839) (Iressa) tablets. Oncologist. 2003;8(4):303-306. doi:10.1634/theoncologist.8-4-303
11. <https://www.accessdata.fda.gov/drugsatfda_docs/label/2010/021743s14s16lbl.pdf>
12. Singh A, Chen H. Optimal Care for Patients with Anaplastic Lymphoma Kinase (ALK)-Positive Non-Small Cell Lung Cancer: A Review on the Role and Utility of ALK Inhibitors. Cancer Manag Res. 2020;12:6615-6628. Published 2020 Jul 30. doi:10.2147/CMAR.S260274
13. Dong J, Li B, Lin D, Zhou Q, Huang D. Advances in Targeted Therapy and Immunotherapy for Non-small Cell Lung Cancer Based on Accurate Molecular Typing. Front Pharmacol. 2019;10:230. Published 2019 Mar 12. doi:10.3389/fphar.2019.00230
14. Anderson KC. Progress and Paradigms in Multiple Myeloma. Clin Cancer Res. 2016;22(22):5419-5427. doi:10.1158/1078-0432.CCR-16-0625
15. Yu C, Liu X, Yang J, et al. Combination of Immunotherapy With Targeted Therapy: Theory and Practice in Metastatic Melanoma. Front Immunol. 2019;10:990. Published 2019 May 7. doi:10.3389/fimmu.2019.00990
16. Lo JA, Fisher DE. The melanoma revolution: from UV carcinogenesis to a new era in therapeutics. Science. 2014;346(6212):945-949. doi:10.1126/science.1253735
17. Garbe C, Eigentler TK, Keilholz U, Hauschild A, Kirkwood JM. Systematic review of medical treatment in melanoma: current status and future prospects. Oncologist. 2011;16(1):5-24.
18. Domingues B, Lopes JM, Soares P, Populo H. Melanoma treatment in review. Immunotargets Ther. 2018;7:35-49
19. Motzer RJ, Russo P. Systemic therapy for renal cell carcinoma. J Urol. 2000;163(2):408-417.
20. Singh A, Singh I, Singh N, Puzanov I. Optimal management of first-line advanced renal cell carcinoma: Focus on pembrolizumab. Onco Targets Ther. 2020;13:4021-4034.
21. <https://grandroundsinurology.com/the-bottom-line-practical-application-of-clinical-trial-data-to-advanced-renal-cell-carcinoma/>
22. Sehn LH, Donaldson J, Chhanabhai M, et al. Introduction of combined CHOP plus rituximab therapy dramatically improved outcome of diffuse large B-cell lymphoma in British Columbia. *J Clin Oncol*. 2005;23(22):5027-5033.
23. Jacobson C, LaCasce A. How I treat Burkitt lymphoma in adults. Blood. 2014;124(19):2913-2920.

# Supplementary Methods: Relative Survival

Five-year relative survival was calculated to assess if differences in survival across time periods influenced tMDS and tAML development. Relative survival compares all-cause overall survival to a “similar” theoretical cancer-free group. Further details regarding relative survival methodology can be found in the supplementary materials. Relative survival is preferable to cause-specific survival due to death certificate misclassification error. The population was restricted to adult survivors (ages 20-84 years) who had survived at least >1-year after first primary NSCLC, cutaneous melanoma, RCC or MM patients diagnosed from 2000-2016 with follow-up through 2017, similar to the previous at-risk analysis. Melanoma and RCC patients were limited to those with advanced disease at diagnosis due to the substantial differences in therapy between early (surgical approaches) and advanced stages (systemic therapies). The same 17 SEER registries were included in addition to the Alaskan registry due to SEER data restrictions. Patients with no survival time and cases identified by death certificate and autopsy only were excluded from analyses. The actuarial method was used to calculate 5-year relative survival, which was investigated across time periods of initial cancer diagnosis (2000-2005, 2006-2010, 2011-2016). Subjects were followed to earliest of: tMDS or tAML diagnosis, last follow-up, age 85 years, death, 5 years, or end of study (December 31, 2017).

| Supplemental Table 2.  Definitions of each cancer by morphology and topography codes | | | |
| --- | --- | --- | --- |
| **Cancer Type** | **Morphology Codes** | **Topography Codes** |  |
| NSCLC | 8003-8004, 8012-8015, 8021-8022, 8030-8035, 8046, 8050-8052, 8070-8076, 8078, 8082-8084, 8090, 8094, 8120, 8123, 8140-8141, 8143-8145, 8147, 8190, 8200-8201, 8211, 8240, 8241, 8243-8246, 8249-8255, 8260, 8290, 8310, 8320, 8323-8333, 8401, 8430, 8440, 8470-8471, 8480-8481, 8490, 8503, 8507, 8525, 8550, 8560, 8562, 8570-8572, 8574-8576 | C340-C349 |  |
| Cutaneous melanoma | 8720-8790 | C440-C449 |  |
| Renal cell carcinoma | all morphology codes excluding 9050-9055, 9140, 9590-9989 | C649 |  |
| Multiple myeloma | 9731-9734 | all topography codes |  |
| tMDS^#^ | 9980, 9982, 9983, 9985, 9986, 9989, 9991, 9992 | all topography codes |  |
| tAML | 9840, 9861, 9865-9867, 9869-9874, 9891, 9895-9898, 9910, 9911, 9920, 9930, 9931, 9984, 9987 | all topography codes |  |
| Abbreviations: NSCLC – non-small cell lung carcinoma, tAML - treatment-related acute myeloid leukemia; tMDS - treatment-related myelodysplastic syndrome.  # Data on tMDS progression to tAML are not available in the SEER database. | | |  |

| Supplemental Table 3.  Risk of tAML by year of first primary diagnosis and initial diagnosis year of 2000-2012 with follow-up through 2017. | | | | | | | | | | | | | | | | | |  |
| --- | --- | --- | --- | --- | --- | --- | --- | --- | --- | --- | --- | --- | --- | --- | --- | --- | --- | --- |
|  |  | Overall | |  |  | 2000-2005 | |  |  | 2006-2010 | |  |  | 2011-2012 | |  |  | |
|  | O | SIR | 95% CI | | O | SIR | 95% CI | | O | SIR | 95% CI | | O | SIR | 95% CI | | Ptrend |  |
| NSCLC | 141 | **2.62*** | **(2.21,** | **3.10)** | 42 | **1.93*** | **(1.39,** | **2.60)** | 75 | **3.38*** | **(2.65,** | **4.23)** | 24 | **2.48*** | **(1.59** | **, 3.69)** | 0.13 | |
| Localized | 43 | **1.79*** | **(1.29** | **, 2.41)** | 14 | 1.41 | (0.77 | , 2.36) | 21 | **2.11*** | **(1.31** | **, 3.22)** | 8 | 1.93 | (0.83 | , 3.80) | >0.20 | |
| Regional | 63 | **3.34*** | **(2.57** | **, 4.27)** | 17 | **2.21*** | **(1.29** | **, 3.54)** | 38 | **4.89*** | **(3.46** | **, 6.71)** | 8 | **2.35*** | **(1.01** | **, 4.63)** | >0.20 | |
| Distant | 32 | **3.36*** | **(2.30** | **, 4.75)** | 10 | **2.89*** | **(1.39** | **, 5.32)** | 16 | **3.92*** | **(2.24** | **, 6.37)** | 6 | **3.02*** | **(1.11** | **, 6.58)** | >0.20 | |
| Unknown | <5 | 2.28 | (0.47 | , 6.67) | <5 | 1.35 | (0.03 | , 7.54) | 0 | **-** | **na** |  | <5 | **12.79*** | **(1.55** | **, 46.18)** | na | |
| Cutaneous melanoma | 66 | 1.13 | (0.88, | 1.44) | 24 | 1.06 | (0.68, | 1.57) | 31 | 1.27 | (0.87, | 1.81) | 11 | 0.99 | (0.49 | , 1.77) | >0.20 | |
| RCC | 49 | 1.13 | (0.94, | 1.68) | 24 | **1.69*** | **(1.08,** | **2.51)** | 20 | 1.18 | (0.72, | 1.83) | 5 | 0.66 | (0.22 | , 1.55) | 0.06 | |
| Multiple myeloma | 75 | **5.59*** | **(4.40,** | **7.00)** | 23 | **4.74*** | **(3.01,** | **7.12)** | 31 | **5.46*** | **(3.71,** | **7.75)** | 21 | **7.26*** | **(4.49** | **, 11.09)** | 0.18 | |
| Abbreviations: CI - confidence interval, NSCLC - non-small cell lung carcinoma, O - observed, RCC - renal cell carcinoma, SIR - standardized incidence ratios, tAML - treatment-related acute myeloid leukemia. | | | | | | | | | | | | | | | | | |  |
| Among adults aged 20-84 years who survived ≥1 year after their first primary cancer diagnosis using 17 Surveillance, Epidemiology, and End Results Program registry areas (Atlanta, Georgia; Connecticut; Detroit, Michigan; Hawaii; Iowa; New Mexico; San Francisco-Oakland, Los Angeles, and San Jose- Monterey, California; Seattle-Puget Sound, Washington; Utah; Kentucky; Louisiana; New Jersey; and areas of Rural Georgia, Greater Georgia, and Greater California) initially diagnosed 2000-2012 with follow-up through 2017. Patients were censored with 5 years of follow-up. | | | | | | | | | | | | | | | | | |  |
| *p<0.05 |  |  |  |  |  |  |  |  |  |  |  |  |  |  |  |  |  | |
| ¥SIRs and 95% confidence intervals compared the number of observed cases with that expected in the general population. | | | | | | | | | | | | | | | | | |  |

| Supplemental Table 4.  Risk of tMDS by year of first primary diagnosis and initial diagnosis year of 2000-2012 with follow-up through 2017. | | | | | | | | | | | | | | | | | |
| --- | --- | --- | --- | --- | --- | --- | --- | --- | --- | --- | --- | --- | --- | --- | --- | --- | --- |
|  |  | Overall |  |  |  | 2000-2005 | |  |  | 2006-2010 | |  |  | 2011-2012 | |  |  |
|  | O | SIR | 95% CI | | O | SIR | 95% CI | | O | SIR | 95% CI | | O | SIR | 95% CI | | Ptrend |
| NSCLC | 141 | **1.96*** | **(1.65,** | **2.31)** | 47 | **1.64*** | **(1.20** | **, 2.18)** | 64 | **2.05*** | **(1.58** | **, 2.62)** | 30 | **2.48*** | **(1.68** | **, 3.55)** | 0.07 |
| Localized | 64 | **1.95*** | **(1.50,** | **2.49)** | 26 | **1.94*** | **(1.27** | **, 2.84)** | 29 | **2.04*** | **(1.37** | **, 2.93)** | 9 | **1.71** | **(0.78** | **, 3.24)** | >0.20 |
| Regional | 37 | **1.48*** | **(1.04,** | **2.04)** | 11 | **1.10** | **(0.55** | **, 1.97)** | 17 | **1.57** | **(0.91** | **, 2.51)** | 9 | **2.13** | **(0.97** | **, 4.05)** | 0.12 |
| Distant | 37 | **3.01*** | **(2.12,** | **4.14)** | 7 | 1.61 | (0.65 | , 3.32) | 18 | **3.22*** | **(1.91** | **, 5.10)** | 12 | **5.05*** | **(2.61** | **, 8.82)** | 0.01 |
| Unknown | <5 | 1.68 | (0.35, | 4.92) | <5 | 3.11 | (0.64 | , 9.10) | 0 | **-** | **na** |  | 0 | **-** | **na** |  | na |
| Cutaneous melanoma | 61 | 0.86 | (0.66, | 1.10) | 22 | 0.80 | (0.50 | , 1.21) | 26 | 0.85 | (0.55 | , 1.24) | 13 | 1.00 | (0.53 | , 1.70) | >0.20 |
| RCC | 51 | 1.06 | (0.79, | 1.39) | 14 | 0.78 | (0.43 | , 1.31) | 26 | 1.20 | (0.79 | , 1.76) | 11 | 1.28 | (0.64 | , 2.28) | >0.20 |
| Multiple myeloma | 69 | **3.94*** | **(3.06,** | **4.98)** | 23 | **3.61*** | **(2.29** | **, 5.42)** | 38 | **4.98*** | **(3.52** | **, 6.83)** | 8 | 2.27 | (0.98 | , 4.47) | >0.20 |
| Abbreviations: CI - confidence interval, NSCLC - non-small cell lung carcinoma, O - observed, RCC - renal cell carcinoma, SIR - standardized incidence ratios, tMDS - treatment-related myelodysplastic syndrome. | | | | | | | | | | | | | | | | | |
| Among adults aged 20-84 years who survived ≥1 year after their first primary cancer diagnosis using 17 Surveillance, Epidemiology, and End Results Program registry areas (Atlanta, Georgia; Connecticut; Detroit, Michigan; Hawaii; Iowa; New Mexico; San Francisco-Oakland, Los Angeles, and San Jose- Monterey, California; Seattle-Puget Sound, Washington; Utah; Kentucky; Louisiana; New Jersey; and areas of Rural Georgia, Greater Georgia, and Greater California) initially diagnosed 2000-2012 with follow-up through 2017. Patients were censored with 5 years of follow-up. | | | | | | | | | | | | | | | | | |
| *p<0.05 |  |  |  |  |  |  |  |  |  |  |  |  |  |  |  |  |  |
| ¥SIRs and 95% confidence intervals compared the number of observed cases with that expected in the general population. | | | | | | | | | | | | | | | | | |

| Supplemental Table 5.  Standardized incidence ratios for tAML/MDS by age, sex, race, initial diagnosis year, and treatment among ≥1-year adult first primary NSCLC, melanoma, RCC, and MM survivors in 17 SEER registries, 2000-2017. | | | | | | | | | | | | | | | | | | | | | | | | | | |
| --- | --- | --- | --- | --- | --- | --- | --- | --- | --- | --- | --- | --- | --- | --- | --- | --- | --- | --- | --- | --- | --- | --- | --- | --- | --- | --- |
| Characteristic |  | NSCLC | |  |  | Cutaneous Melanoma | | | | |  | |  |  | | RCC | |  |  | |  | |  | MM | |  |
|  |  | n=206,608 | |  |  |  |  | n=191,793 | |  |  | |  |  | | n=121,492 | |  |  | |  | |  | n=45,256 | |  |
|  | O | SIR | 95% CI | |  | O |  | SIR | 95% CI | |  | | O |  | | SIR | 95% CI | |  | | O | |  | SIR | 95% CI | |
| Overall | 363 | 2.38* | (2.14, | 2.63) |  | 157 |  | 1.00 | (0.85, | 1.17) |  | | 118 |  | | 1.12 | (0.93, | 1.34) |  | | 172 | |  | 4.46* | (3.81, | 5.17) |
| Age at first primary neoplasm, years | | | | | | | | | | | | | | | | | | | | | | | | | | |
| <50 | 13 | 9.94* | (5.29, | 16.99) |  | 6 |  | 0.86 | (0.31, | 1.86) | [ | | 18 | ] | | 1.27 | (0.76, | 2.01) |  | | 6 | |  | 10.02* | (3.68, | 21.81) |
| 50-59 | 43 | 4.74* | (3.43, | 6.39) |  | 21 |  | 1.29 | (0.80, | 1.97) |  |  |  |  |  |  |  |  |  | | 36 | |  | 11.38* | (7.97, | 15.75) |
| 60-69 | 108 | 2.50* | (2.05, | 3.02) |  | 51 |  | 1.15 | (0.86, | 1.51) |  | | 38 |  | | 1.14 | (0.81, | 1.57) |  | | 69 | |  | 6.13* | (4.77, | 7.76) |
| 70-79 | 175 | 2.10* | (1.80, | 2.44) |  | 68 |  | 0.92 | (0.72, | 1.17) |  | | 49 |  | | 0.99 | (0.73, | 1.31) |  | | 51 | |  | 2.61* | (1.95, | 3.44) |
| ≥80 | 24 | 1.51 | (0.96, | 2.24) |  | 11 |  | 0.70 | (0.35, | 1.26) |  | | 13 |  | | 1.57 | (0.84, | 2.68) |  | | 10 | |  | 2.45* | (1.17, | 4.51) |
| Ptrend |  | < 0.001 | |  |  |  |  | 0.20 | |  |  | |  |  | | >0.20 | |  |  | |  | |  | < 0.001 | |  |
| Sex |  |  |  |  |  |  |  |  |  |  |  | |  |  | |  |  |  |  | |  | |  |  |  |  |
| Male | 210 | 2.30* | (2.00, | 2.63) |  | 108 |  | 0.94 | (0.77, | 1.14) |  | | 80 |  | | 1.06 | (0.84, | 1.31) |  | | 112 | |  | 4.41* | (3.63, | 5.30) |
| Female | 153 | 2.50* | (2.12, | 2.92) |  | 49 |  | 1.16 | (0.86, | 1.53) |  | | 38 |  | | 1.30 | (0.92, | 1.78) |  | | 60 | |  | 4.55* | (3.47, | 5.86) |
| Pheterogeneity |  | >0.20 | |  |  |  |  | >0.20 | |  |  | |  |  | | >0.20 | |  |  | |  | |  | >0.20 | |  |
| Race |  |  |  |  |  |  |  |  |  |  |  | |  |  | |  |  |  |  | |  | |  |  |  |  |
| White/Unknown | 298 | 2.23* | (1.98, | 2.50) |  | 155 |  | 0.99 | (0.84, | 1.16) |  | | 95 |  | | 1.03 | (0.83, | 1.26) |  | | 141 | |  | 4.53* | (3.82, | 5.35) |
| Black | 39 | 3.57* | (2.54, | 4.88) | [ | <5 | ] | 1.68 | (0.20, | 6.08) |  | 16 | | |  | 1.94* | (1.11, | 3.16) | |  | | 23 |  | 4.00* | (2.54, | 6.00) |
| Other | 26 | 3.17* | (2.07, | 4.64) |  |  |  |  |  |  |  | 7 | | |  | 1.59 | (0.64, | 3.28) | |  | | 8 |  | 4.59* | (1.98, | 9.05) |
| Pheterogeneity |  | 0.09 | |  |  |  |  | na | |  |  | |  |  | | 0.11 | |  |  | |  | |  | >0.20 | |  |
| Initial diagnosis year |  |  |  |  |  |  |  |  |  |  |  | |  |  | |  |  |  |  | |  | |  |  |  |  |
| 2000-2005 | 89 | 1.76* | (1.41, | 2.16) |  | 46 |  | 0.92 | (0.67, | 1.22) |  | | 38 |  | | 1.18 | (0.83, | 1.62) |  | | 46 | |  | 4.09* | (3.00, | 5.46) |
| 2006-2010 | 139 | 2.59* | (2.18, | 3.06) |  | 57 |  | 1.04 | (0.78, | 1.34) |  | | 46 |  | | 1.19 | (0.87, | 1.59) |  | | 69 | |  | 5.17* | (4.02, | 6.54) |
| 2011-2015 | 135 | 2.78* | (2.33, | 3.29) |  | 54 |  | 1.04 | (0.78, | 1.36) |  | | 34 |  | | 0.99 | (0.69, | 1.39) |  | | 57 | |  | 4.07* | (3.08, | 5.27) |
| Ptrend |  | < 0.001 | |  |  |  |  | >0.20 | |  |  | |  |  | | >0.20 | |  |  | |  | |  | >0.20 | |  |
| Chemotherapy |  |  |  |  |  |  |  |  |  |  |  | |  |  | |  |  |  |  | |  | |  |  |  |  |
| No/unknown chemotherapy | 171 | 1.69* | (1.45, | 1.97) |  | 152 |  | 0.97 | (0.83, | 1.14) |  | | 115 |  | | 1.13 | (0.94, | 1.36) |  | | 44 | |  | 2.80* | (2.04, | 3.76) |
| Any chemotherapy | 192 | 3.74* | (3.23, | 4.31) |  | 5 |  | 4.30* | (1.40, | 10.03) |  | | <5 |  | | 0.89 | (0.18, | 2.61) |  | | 128 | |  | 5.61* | (4.68, | 6.67) |
| Pheterogeneity |  | < 0.001 | |  |  |  |  | na | |  |  | |  |  | | na | |  |  | |  | |  | 0.001 | |  |
| Radiation |  |  |  |  |  |  |  |  |  |  |  | |  |  | |  |  |  |  | |  | |  |  |  |  |
| No/unknown radiation | 188 | 1.72* | (1.48, | 1.99) |  | 153 |  | 0.99 | (0.84, | 1.15) |  | | 115 |  | | 1.11 | (0.92, | 1.34) |  | | 145 | |  | 4.45* | (3.75, | 5.23) |
| Any radiation | 175 | 4.05* | (3.47, | 4.70) |  | <5 |  | 2.28 | (0.62, | 5.83) |  | | <5 |  | | 2.06 | (0.43, | 6.03) |  | | 27 | |  | 4.59* | (3.03, | 6.68) |
| Pheterogeneity |  | < 0.001 | |  |  |  |  | na | |  |  | |  |  | | na | |  |  | |  | |  | >0.20 | |  |
| 17 SEER registries (Atlanta, Connecticut, Detroit, Hawaii, Iowa, New Mexico, San Francisco-Oakland, Seattle-Puget Sound, Utah, Los Angeles, San Jose- Monterey, Rural Georgia, Greater California, Kentucky, Louisiana, New Jersey, and Greater Georgia), diagnosed 2000-2016 with follow up through 2017, attained age<85 years, 12-month survivors. | | | | | | | | | | | | | | | | | | | | | | | | | | |
| Abbreviations: MM - multiple myeloma; NSCLC - non-small cell lung carcinoma; O - observed; RCC - renal cell carcinoma; SEER - Surveillance, Epidemiology  and End Results Program; SIR - standardized incidence ratio; tAML - treatment-related acute myeloid leukemia; 95% CI - 95% confidence interval. | | | | | | | | | | | | | | | | | | | | | | | | | | |
| * p<0.05. | | | | | | | | | | | | | | | | | | | | | | | | | | |
| Note: P values to test differences in the SIRs were computed using a likelihood ratio test derived from Poisson regression models stratified by age at first  primary neoplasm, sex, race, initial diagnosis year and stage of NSCLC. Categories with <5 observations were not specified to maintain patient confidentiality.  [n] indicate categories with <5 that were grouped for analyses. | | | | | | | | | | | | | | | | | | | | | | | | | | |

Figure 1. Population-based patterns of decreasing chemotherapy use and adoption of modern therapies for non-small cell lung cancer, cutaneous melanoma, renal cell carcinoma and multiple myeloma before and after year 2000

**NSCLC:**

2003: TKIs for advanced disease

2011: ICI in trials for advanced disease

**Melanoma**:

1995: IL-2, IFN-α and Dacarbazine

2011: BRAF- TKI and ICI

**RCC:**

Early 2000s: Chemo use declined

2005: VEGF TKI approval

2011-2016: ICI

**MM:**

2007: Lenalidomide use ↑

Abbreviations: NSCLC- Non-small cell lung cancer; TKI – Tyrosine Kinase Inhibitors; IL2 – Interleukin 2; IFN-α – Interferon alpha; BRAF- BRAF inhibitor; ICI – Immune checkpoint inhibitor; RCC – Renal Cell Carcinoma; VEGF – Vascular Endothelial Growth Factor; MM – Multiple myeloma
